# Genetic variability in E6 and E7 oncogenes of human papillomavirus Type 16 from Congolese cervical cancer isolates

**DOI:** 10.1186/s13027-015-0010-4

**Published:** 2015-05-14

**Authors:** Luc Magloire Anicet Boumba, Samira Zoa Assoumou, Lahoucine Hilali, Jean Victor Mambou, Donatien Moukassa, Mustapha Moulay Ennaji

**Affiliations:** Laboratoire de Virologie, Microbiologie et Qualité/ETB, Faculté des Sciences et Techniques de Mohammedia, Université Hassan II Casablanca, B.P. 146, 20650 Mohammedia, Maroc; Laboratoire d’Agroalimentaire et Santé, Département de Biologie Appliquée, Faculté des Sciences et Techniques, Université Hassan 1er Settat B.P. 577 Settat, Mohammedia, Maroc; Laboratoire d’Analyses Médicales et Morphologiques, Hôpital Général de Loandjili, B.P. 8122, Pointe-Noire, Congo; Centre Médico-social de la Mairie Centrale de Pointe-Noire, B.P. 383, Place de la Liberté, 97116 Pointe-Noire, Congo

**Keywords:** Human papillomavirus type 16, E6 and E7 genetic variants, Southwest Congo

## Abstract

**Background:**

The molecular epidemiological studies showed that some variants of HPV-16, distributed geographically, would present a higher risk of causing cervical cancer. This study aimed to analyze nucleotide changes of HPV-16 E6 and E7 genomic regions from infected Southwestern Congolese women.

**Methods:**

DNA of twenty HPV-16 isolates was analyzed by amplifying the E6 and E7 genes using type-specific primers PCR and direct sequencing. The sequences obtained were aligned with the HPV-16 GenBank reference sequences.

**Results:**

Thirteen (65.0%) out of 20 DNA-samples were successfully amplified. Genetic analysis revealed 18 and 4 nucleotide changes in E6 and E7 genomic regions respectively. The most frequently observed nucleotide variations were the missense C143G, G145T and C335T in E6 (100%), leading to the non-synonymous amino acid variation Q14D and H78Y. E7 genomic region was found to be highly conserved with two most common T789C and T795G (100%) silent variations. All HPV-16 variants identified belonged to the African lineage: 7 (53.8%) belonged to Af-1 lineage and 6 (46.1%) to Af-2 lineage. The missense mutation G622A (D21N) in the E7 region seems to be described for the first time in this study.

**Conclusion:**

This study reported for the first time the distribution of HPV-16 E6 and E7 genetic variants in infected women from southwest Congo. The findings confirmed almost ascendancy of the African lineage in our study population.

## Background

The Republic of Congo has one of the highest rates of cervical cancer incidence in Sub-Sahara Africa. According to the World Health Organization (WHO), the reported age-standardized incidence rate and age-standardized mortality rate were 25.6 and 13.0 cases per 100 000 women in 2014 [1].

Epidemiological and molecular studies are showed that High-risk human papillomaviruses (HPV) types are the etiological agents of cervical cancer [2,3]. HPV-16 is the most common high-risk genotype involved in cervical cancer, representing for over 50% of all cases [4].

However, most HPV infections resolve spontaneously, suggesting that other co-factors are necessary in the persistence and lesion progression to cancer. These cofactors include some variants of high-risk types such as those of HPV16. Several molecular variants were described and classified according to geographical regions for the HPV-16 genotype [5,6]. Conventionally, a variant is defined when there is a difference of less than 2% between the complete genomes of the same HPV type. A difference from 1.0% has been used to define the lineage and the sub-lineages variants by a difference from 0.5 to 1.0% between the lineages [7-9]. The variants have been classified in six major phylogenetic lineages (European, E; Asian, As; African-1, Af-1; African-2, Af-2; Asian-American, AA and North-American, NA) showing more than 98% nucleotide level similarity in L1 or E6 genes [10,11]. However, a new phylogenetic nomenclature was proposed for the HPV-16 in four lineages as follows: A (previously called European-Asian, EAS), B (African 1, Af-1), C (African 2, Af-2), and D (North-American/Asian-American, NA/AA) [7,9].

HPV-16 variants studies have shown that certain intratypic-variants would contribute more than others to the persistence of HPV infection and cervical cancer progression [12,13]. In particular, non-European variants are more highly oncogenic them European variants [12,14,15]. Indeed, the importance of the E6 and E7 genes lies in their ability to transform and immortalize the host cell. A single nucleotide change in these genes could affect this ability, which would explain the difference in some variants to progress quickly them others to cervical cancer [16]. E6 and E7 interacts with several cellular proteins in particular, they inactivate the p53 and the pRb respectively, which are two cancer suppressor major proteins [17].

In this perspective and with the advent of prophylactic vaccines against HPV-16/18 and cervical cancer, it becomes necessary to know the HPV genetic variants present in each population.

In Congo, our previous study showed that the most common genotype in invasive cervical cancer and its precancerous lesions was HPV-16 [18]. Thus, to better formulate immunization strategies and other preventive measures in Congo, this preliminary study aimed to analyze the nucleotide changes of the HPV-16 E6 and E7 isolates in order to identify the different genetic variants prevalent among infected women with cervical carcinoma in southwest of the Congo.

## Results

Out of 20 HPV-16 single infected cervical cancer samples examined in this study, the E6 and E7 genes were able to be amplified and sequenced in 13 (13/20, 65.0%) DNA samples. Nucleotide (nt) changes were analyzed between nt 104 to 559 for E6 region and nt 562 to 858 for E7 region.

### E6 nucleotide sequences analysis

By comparison with the HPV-16 reference sequence (European prototype, NC_001526), mutation analysis showed that 100% (13/13) of the analyzed sequences contained at least one nucleotide change in the E6 region. Eighteen single nucleotide changes were observed in HPV-16 E6 sequences, of which 13/18 (72.2%) were non-synonymous mutations (all missense) and 5/18 (27.8%) were synonymous mutations (silent). The most frequently observed variations were C143G (Q14D), G145T (Q14D), T286A, A289G and C335T (H78Y), which was found in all samples. All nucleotide changes, variants and their prevalence were reported in Table 1.

**Table 1:** **Nucleotide sequence variations of HPV-16 E6 and E7 genes, predicted amino-acid changes ant lineage classification in Southwest Congolese women**

				**E6 nucleotide position**																	**E7 nucleotide position**			
			**Amino acid changes**		**R10G**	**R101/R10T**	**L12I**	**L12S**	**P13G**		**Q14D**	**Q14H**	**E29Q**	**C51F**	**A6IG**			**D64E**	**H78Y**		**D2IN**	**N29S**		
				1	1	1	1	1	1	1	1	1	1	2	2	2	2	2	3	4	6	6	7	7
				0	3	3	3	3	4	4	4	4	8	5	8	8	8	9	3	0	2	4	8	9
				9	1	2	7	8	1	2	3	5	8	5	5	6	9	5	5	3	2	7	9	5
			HPV16 Ref (NC_001526)	**T**	**A**	**G**	**T**	**T**	**C**	**A**	**C**	**G**	**G**	**G**	**C**	**T**	**A**	**T**	**C**	**A**	**G**	**A**	**T**	**T**
Variant Lineages	Variant Sub-lineages	Novel nomenclature	Prevalence																					
			No. (%)																					
Af-1	Af-la	B1	4 (30.7)			C					g	G	T	C		a	g		T				c	g
	Af-Ib/G295	B2	1 (7.7)			C						G	T			a	g	G	T				c	g
	Af-Ib/G131	B2	2 (15.4)		G	C						G	T			a	g		T				c	g
Af-2	Af-2a/r	C/C1	3 (23.0)	c		T					G	T				a	g		T	g	A*		c	g
	Af-2a/C109/C138		1 (7.7)	c		T		C			G	T				a	g	G	T	g		G	c	g
	Af-2a/C109/A137		1 (7.7)	c		T	A		G		G	T				a	g		T	g		G	c	g
	Af-2a/C109/G285		1 (7.7)	c		T					G	T			G	a	g		T	g		G	c	g
			Mutation prevalence (%)	46.1	15.4	53.8/46.1	7.7	7.7	7.7	7.7	100	100	30.7	15.4	7.7	100	100	7.7	100	46.1	23.0	23.0	100	100

Capital letters indicate variants with an amino acid change, Lower-case letters indicate silent mutations, M, missense; S, silence; The asterisk (*) indicates mutations never described.

### E7 nucleotide sequences analysis

The details of E7 mutations are summarized in Table 1. E7 gene was found to be highly conserved in all cases compared to E6 region. Only four single nucleotide changes were observed in HPV-16 E7 sequences, with 2/4 (50.0%) missense and 2/4 (50.0%) silent. The most frequently observed variations were the common silent mutations T789C and T795G at codons 76 and 78, which was found in all E7 sequences. However, the common non-synonymous mutation A to G transition at nt 647 and causing amino acid change N29S, was found in 3 (23.0%) out of 13 sequences. An undescribed mutation was found in three sequences in this study at nt 622, that was generated a non-synonymous nucleotide change G622A (D21N).

### HPV-16 variants and phylogenetic analysis

**Figure 1:**
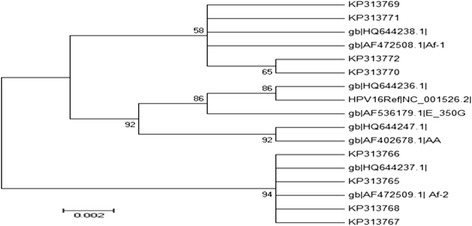
Maximum Likelihood Molecular Phylogenetic analysis using eight nucleotide sequences of HPV-16 E6 gene. Study sequences are labeled in KP GenBank accession numbers. Others are reference GenBank sequences, including: NC_001526 (HPV-16 Ref), HQ644236, AF536179, HQ644238, AF472508, HQ644237, AF472509, HQ644247, AF402678 and HQ644292. Phylogenetic trees were constructed by the Maximum Likelihood method and the Kimura 3-Parameter model by MEGA package. Bootstrap proportions were calculated with 1000 replicates.

**Figure 2:**
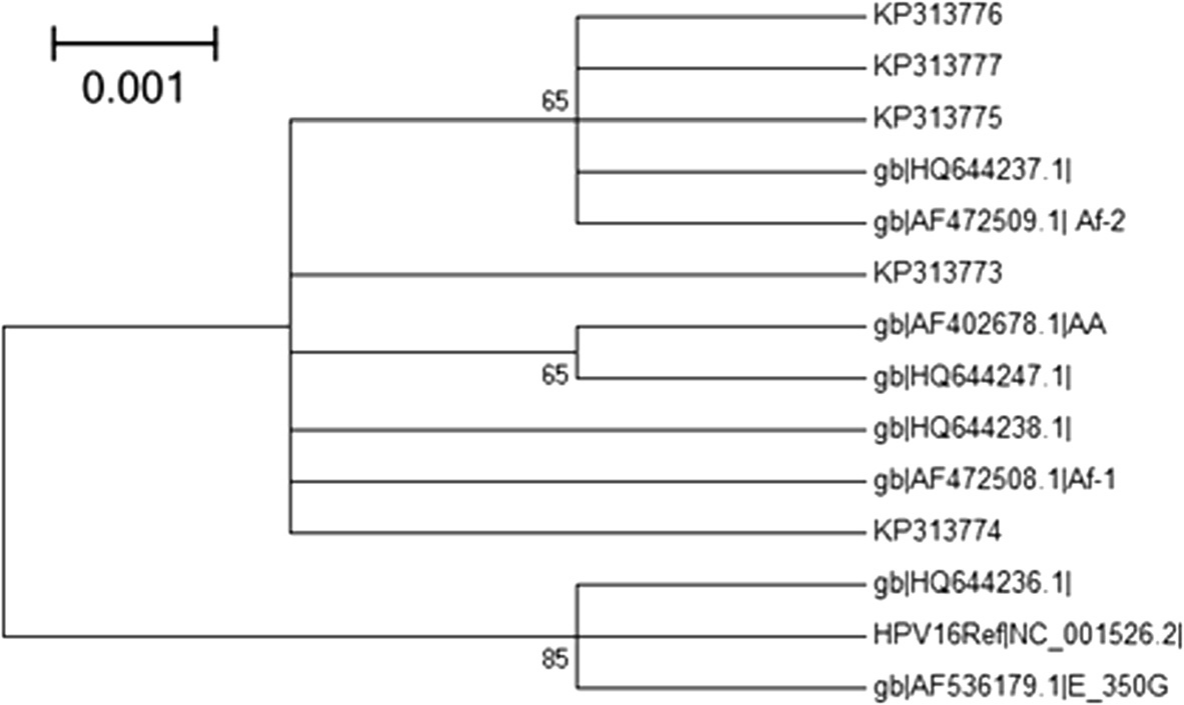
Maximum Likelihood Molecular Phylogenetic analysis using five nucleotide sequences of HPV-16 E7 gene. Study sequences are labeled in KP GenBank accession numbers. Others are reference GenBank sequences, including: NC_001526 (HPV-16 Ref), HQ644236, AF536179, HQ644238, AF472508, HQ644237, AF472509, HQ644247, AF402678 and HQ644292. Phylogenetic trees were constructed by the Maximum Likelihood method and the Kimura 3-Parameter model by MEGA package. Bootstrap proportions were calculated with 1000 replicates.

All HPV-16 positive sequences analyzed belonged to the African lineage, of which 7 (53.8%) out of 13 belonged to Africain-1 lineage (Af-1) and 6 (46.1%) out of 13 to Africain-2 lineage (Af-2). All African lines showed a common pattern of five characteristic mutations in E6, namely, C143G, G145T, T286A, A289G, and C335T, which lead to two non-synonymous amino acid changes Q14D and H78Y. In E7, all HPV-16 isolates showed a common pattern of two silent mutations namely T789C and T795G at codons 76 and 78 respectively. No European (E), Asian (As), Asian-American (AA) or northern American (NA) lineages were detected in this study. Phylogenetic trees were respectively built from eight sequences E6 regions (Figure 1) and 5 of the E7 region (Figure 2) from this study and other HPV-16 published sequences available in GenBank.

## Discussion

The HPV-16 genotype was recently identified as the most prevalent genotype in cervical cancer cases and high grade precancerous lesions in the southwest of Congo [18].

To date, there are no molecular epidemiological studies on HPV-16 variants carried out in Congo. In the present study, we characterized the nucleotide changes of the HPV16 mono-infection isolates among infected Congolese women with cervical cancer based on the E6 and E7 viral oncogenes analysis.

Several studies showed that HPV-16 variants may influence viral persistence and progression to cervical cancer, which partly explains the high prevalence of this cancer in some populations compared to other [12]. Also, their distribution is geographically related and associated with the ethnic group [19,20]. The HPV 16 E6 and E7 early genes are essential in cervical cancer pathogenesis [21]. Any change in their sequences could induce an alteration of the biological function of the proteins encoded by these genes, which would justify the importance of these kinds of studies, especially in the poorly studied populations as ours.

In this study, phylogenetic analysis of HPV16 E6 and E7 genes was performed to analyze the local distribution of HPV16 lineages, according to the Yamada et al. [5] classification. A total of eighteen point mutations in E6 and four in E7 genomic regions were found. Most nucleotide changes reported in our study have been previously described. At least a nucleotide change was observed in all sequences analyzed. This result is consistent with the literature data indicating that more than 90% of the E6 sequences contains mutations in cervical carcinomas, whereas the E7 gene seems to be more conserved [5,6,22,23].

At the E6 genomic region, five characteristic mutations, C143G, G145T, T286A, A289G, and C335T defining the African lineage were found in our study as well as it’s was previously described [8,11,24]. However, some of the observed mutations are significant in the literature; in particular the two non-synonymous changes African lineage located in E6 N-terminus coding region at codon 10 and 14, which lead to amino acid changes R10T and Q14D respectively. These amino acid changes may lead to a difference in affinity with p53 and significantly altered its degradation rate by this oncogene [25,26]. Crook et al. [27] showed that the amino acid mutations in these positions increased by 180% the affinity of E6 with p53. It is also reported that the amino acid change R10I in addition to other mutations described between nucleotide position 106 and 113 could lead to a low affinity for binding of E6 and degrade p53 [25], although these findings remain controversial and poorly documented.

In contrast to E6, the E7 gene variability has been less studied. Our studies on E7 showed a highly conserved region of the HPV genome with some mutations (mainly point mutations) described and few lead to amino acid changes [23,28]. Out of four mutations identified in our study, two were the common silent variations found in all sequences. The two others were non-synonymous mutations, of which a common described N29S was found in three isolates (23%). This mutation often described in Asia, was located in the cervical carcinoma in Africa [23,29,30]. The importance of this amino acid change was suggested by Zehbe et al. [22] because of its location in an immunoreactive region. However, E7 protein with the N29S mutation present an identical power transforming has the prototype strain, after measuring the cooperation E7/activated *ras* gene in rat embryo fibroblast cells [31]. In addition, N29S is involved in binding to pRB and could alter the affinity of the E7 protein for pRB, or modification of the oncogenic potential. But it was also proved that the variant A647G only binds with similar affinity to that of the prototype [32,33]. The D21N mutation (G622A) described in this study seems to be for the first time. Only one case showed this variation; future studies by sequencing after cloning in a vector are needed to confirm its description in our population.

All HPV-16 isolates examined in this study belonged to the African lineage, of which 53.8% belonged to Af-1 lineage and 46.1% to Af-2 lineage. Our results are in agreement with the literature. Indeed, the pioneering studies of Yamada and Wheeler showed that the African lineage was dominated in the continent. More than 90.7% of the variants in Africa region are African lineage, of which 61% belongs to the Af-1 lineage and 29.7% to the Af-2 lineage [6,11].

Tu et al. [34] in South Africa, Qmichou et al. [35] in Morocco and Buonaguro et al. [30] in Uganda reported have identified in their respective studies 41%, 64.5% and 100% of variants belonging to the African lineage. These results substantially similar show the importance of taking into account the African variants in the global effort to fight against cervical cancer. The findings obtained by Qmichou et al. [30] which reported 35.5% of variants belonged to the Af-1 lineage and 29% to the Af-2 lineage such as Yamada (61% Af-1 and 29.7% Af-2) are consistent with ours by showing that the Af-1 lineage is predominant on the continent [11]. However, non-African lineages were also reported in the continent, especially in North Africa and South Africa regions. The introduction of these lineages could be explained by the diversity of their population with European populations. In our study, the absence of non-African lineages could be explained by the small size of our sample, but also by the fact that their frequency is very low in a homogeneous African black population. However, further investigation seems to be necessary to confirm this hypothesis.

Knowing that HPV variants data are important in developing HPV diagnostics, vaccines, and other therapeutic approaches to monitoring virus-induced diseases [28], the major limitation of this study was the small size of DNA-sample analyzed. However, this is the first study that provides information on HPV-16 E6 and E7 genetic diversity in Congolese women. A larger sample size of HPV 16 including E6 and E7, but also L1 and LCR genes will be necessary to better identify all the genomic changes and appreciate their impact on the various grades of cervical lesions.

## Conclusion

The present study provides preliminary data on HPV16 E6 and E7 variants in the southwest of Congo. Findings confirmed the predominance of the African lineage in our samples, which could guide control strategies against cervical cancer in Congo. However, future studies with a large sample are needed to better assess the distribution of these variants depending on the grade and the risk of developing cervical cancer.

## Materials and methods

### DNA Biological samples

DNA of twenty HPV-16 single infection isolates of Congolese women with cervical cancer from our laboratory DNA bank (Laboratory of Virology, Microbiology and Quality/ETB, Faculty of Sciences and Techniques de Mohammedia, UH2C, Morocco) constituted from our previous study [18] was investigated. In brief, All the samples of biopsies were squamous cell carcinoma (SCC) selected based on the availability of the DNA sample. Samples were collected from the patients attending the pathology laboratory of the General Hospital of Loandjili (Pointe-Noire, Southwest Congo).

### PCR amplification with type-specific (TS) primers and sequencing

Amplification of HPV-16 E6 and E7 genes was performed using TS primers flanking the encoding regions of HPV16 E6 ORF (nt: 41–576) and HPV16 E7 ORF (nt: 483–911), as previously described [36]. PCR was performed with some minor modifications in 25 μL of reaction mixture containing 1X PCR buffer, 2.5 mM MgCl_2_, 0.8 mM of each dNTP, 0.4 μM/μL of sense and antisense primers, 0.025U/μl of Go Taq DNA polymerase (Promega) and 1 μL of template DNA. The thermal program (Perkin Elmer 2400 GeneAmp® PCR thermal Cycler, Scientific Support, Inc, Hayward, CA) started with a pre-heat of 94°C for 10 min, followed by 35 cycles according to the protocol: 45 sec denaturation at 94°C, 45 sec annealing at 49.5°C for E6 and 53.5°C for E7 and 45 sec extension at 72°C, with 7 min final elongation at 72°C. For each reaction, DNA from SiHa cell lines as positive controls and Ultra-pure PCR water nuclease-free (Bioline, UK) as negative PCR control were included.

PCR products were analyzed based on the expected specific bands in 2% agarose gel. Type-specific PCR products were directly sequenced using the “Big Dye Terminator v3.1 Cycle Sequencing kit” (Applied Biosystems) according to the manufacturer’s instructions in molecular and functional genomics platform (UATRS-CNRST, Rabat, Morocco).

### Phylogenetic analysis of sequences

The sequences were subsequently analyzed by NCBI using online BLAST 2.0 software server (http://www.ncbi.nih.gov/BLAST/) to confirm genotype and amplified region. Multiple sequence alignments of the full-length of E6 and E7 genes were performed using BioEdit Sequence Alignment Software v7.0.4.1 and Clustal W program [37]. HPV-16 DNA nucleotide positions were numbered according to the HPV-16 reference (European prototype published in GenBank with accession number: NC_001526.2) [38].

Phylogenetic trees were built by MEGA 5.1 package [39] using a distance-based criterion, Tamura 3-parameter as the substitution model [40] and Maximum Likelihood algorithm with bootstrap proportions were calculated with 1000 replicates to test the robustness of the major phylogenetic groups [41]. The reference viral strains used for the construction of phylogenetic trees were obtained from the NCBI GenBank Database, which belong to Asian-American lineage (GenBank accession numbers: AF402678; HQ644247), African-1 (GenBank: AF472508; HQ644238), African-2 (GenBank: AF472509; HQ644237) lineage, and European lineage (GenBank: AF536179; HQ644236).

### GenBank accession numbers

The representative nucleotide sequences from each region sequenced in this study were deposited into GenBank Database (http://www.ncbi.nlm.nih.gov/GenBank), under the accession numbers KP313765 to KP313772 for E6 and KP313773 to KP313777 for E7 genes.

### Ethics statement

During the collection of samples that have been the subject of this study, informed consent was obtained for all women and the study protocol was approved by the local ethical review board of research in health Sciences of Congo ("Comité d’éthique de la Recherche en Sciences de la Santé").
